# Enhanced notch signaling modulates unproductive revascularization in response to nitric oxide‐angiopoietin signaling in a mouse model of peripheral ischemia

**DOI:** 10.1111/micc.12549

**Published:** 2019-06-19

**Authors:** Maria J. C. Machado, Rachel Boardman, Federica Riu, Costanza Emanueli, Andrew V. Benest, David O. Bates

**Affiliations:** ^1^ Division of Cancer and Stem Cells Tumour and Vascular Biology Laboratories, Cancer Biology School of Medicine Queen's Medical Centre University of Nottingham Nottingham UK; ^2^ National Heart & Lung Institute Imperial College London London UK; ^3^ COMPARE University of Birmingham and University of Nottingham Nottingham UK

**Keywords:** arteriolargenesis, delta‐like ligand 4, hindlimb ischemia, NO‐Tie, vascular endothelial growth factor

## Abstract

**Introduction:**

Arteriolargenesis can be induced by concomitant stimulation of nitric Oxide (NO)‐Angiopoietin receptor (Tie)‐Vascular Endothelial Growth Factor (VEGF) signaling in the rat mesentery angiogenesis assay. We hypothesized that the same combination of exogenously added growth factors would also have a positive impact on arteriolargenesis and, consequently, the recovery of blood flow in a model of unilateral hindlimb ischemia.

**Results and Methods:**

NO‐Tie mice had faster blood flow recovery compared to control mice, as assessed by laser speckle imaging. There was no change in capillary density within the ischemic muscles, but arteriole density was higher in NO‐Tie mice. Given the previously documented beneficial effect of VEGF signaling, we tested whether NO‐Tie‐VEGF mice would show further improvement. Surprisingly, these mice recovered no differently from control, arteriole density was similar and capillary density was lower. Dll4 is a driver of arterial specification, so we hypothesized that Notch1 expression would be involved in arteriolargenesis. There was a significant upregulation of Notch1 transcripts in NO‐Tie‐VEGF compared with NO‐Tie mice. Using soluble Dll4 (sDll4), we stimulated Notch signaling in the ischemic muscles of mice. NO‐Tie‐sDll4 mice had significantly increased capillary and arteriole densities, but impaired blood flow recovery.

**Conclusion:**

These results suggest that Dll4 activation early on in revascularization can lead to unproductive angiogenesis and arteriolargenesis, despite increased vascular densities. These results suggest spatial and temporal balance of growth factors needs to be perfected for ideal functional and anatomical revascularisation.

**Abbreviations**
Ang1: Angiopoietin 1
CHO: Chinese hamster ovary
Dll4: delta‐like ligand 4
ECs: endothelial cells
IB4: isolectin B4
MOI: multiplicity of infection
NICD: notch intracellular domain
NO: nitric oxide
PAD: peripheral arterial disease
VEGF: vascular endothelial growth factor
VSMCs: vascular smooth muscle cells

## 1 INTRODUCTION

PAD has become a global problem in the 21st century and is a common morbidity factor associated with some chronic diseases, such as diabetes.^1^ Although amputation‐free survival has steadily increased in high income countries, retrospective long‐term follow‐up studies reveal that the severe metabolic damage caused by ischemia still leads to poor prognosis in critical limb ischemia patients.^2^ Treatment of PAD relies upon effective vascular remodeling resulting in a “vascular tree,” comprising the coordinated induction of arteriole, venule, and capillary identities that are capable of controlling blood flow. Moreover, regulated perfusion requires the formation of muscularized conduit vessels to deliver sufficient blood flow to the capillary exchange vessels.

The early promise of preclinical trials using VEGF as a therapeutic did not give rise to the expected results.^3^ Angiogenesis induced by VEGF over‐expression is abnormal as vessels lack pericytes or VSMCs, appear thin walled and hypertrophic, and are prone to regression upon VEGF withdrawal.^4^, ^5^, ^6^ To develop a mature, stable microvasculature, other proteins appear to be necessary.^7^

As simply over‐expressing individual growth factors has demonstrated that some, but not all aspects of therapeutic revascularization can be achieved, it seems likely that the spatial, temporal, and biochemical co‐ordination of specific growth factors will be key to effective vascular remodeling. As such, studies combining delivery of different growth factors have started to report more encouraging results. For example, combining VEGF_165_ and Ang1 improved the outcome in a diabetic rat model of myocardial infarction by reducing fibrosis and ventricular remodeling, along with increased angiogenesis.^8^ It has also been shown, in a rat dorsal skin flap model that the mechanism through which adenoviral‐mediated delivery of VEGF (through Ad.VEGF) increases tissue viability and blood flow is due to an increase in NO synthesis and release.^9^

Using a model of physiological angiogenesis, the rat mesentery assay, we previously described the process of arteriolargenesis, the generation of arterioles from capillaries.^10^, ^11^, ^12^ We have shown that, by over‐expressing eNOS (endothelial nitric oxide synthase), we promote a NO‐mediated increase in blood flow, which gives rise to angiogenesis dependent upon VEGF‐VEGFR and Ang1‐Tie signaling,^12^ which was more beneficial than the combination of Ang1+VEGF alone.^11^ Furthermore, the combination of adenoviral‐mediated delivery of eNOS and Ang1 (NO‐Tie) is sufficient to promote arteriolargenesis,^12^ but to a reduced degree compared with adenovirally mediated delivery of eNOS, Ang1, and VEGF (NO‐Tie‐VEGF).^10^

VEGF has been shown to induce activation of the Dll4 notch pathway during neovascularization. Dll4 activation results in less permeable blood vessels, and a more mature vasculature, with more arterioles and fewer branch points. We therefore tested the hypothesis that adding in Dll4 activation to the NO‐Tie‐VEGF combination might generate an improved revascularization.

## 2 MATERIALS AND METHODS

### 2.1 Viruses

Human embryonic kidney cells (HEK293) and CHO cells were purchased from ATTC and used to expand or test all adenoviruses used.

Ad.eGFP (enhanced green fluorescent protein, used as control) and Ad.Ang1* (Angiopoietin 1) were a gift from Regeneron Inc., Tarrytown, USA.^12^, ^13^ Ad.eNOS (endothelial nitric oxide synthase) was a gift from Prof Keith Channon and gave rise to increased production of nitric oxide.^12^, ^13^ Ad.VEGF was previously characterized as giving rise to the over‐expression of the human VEGF‐A_165_a isoform in the mesentery.^6^

Ad.sDll4 was custom designed and purchased from Vector Biolabs. The sequence of the sDll4 was as follows:

MTPASRSACR WALLLLAVLW PQQRAAGSGI FQLRLQEFVN QRGMLANGQS CEPGCRTFFR

ICLKHFQATF SEGPCTFGNV STPVLGTNSF VVRDKNSGSG RNPLQLPFNF TWPGTFSLNI

QAWHTPGDDL RPETSPGNSL ISQIIIQGSL AVGKIWRTDE QNDTLTRLSY SYRVICSDNY

YGESCSRLCK KRDDHFGHYE CQPDGSLSCL PGWTGKYCDQ PICLSGCHEQ NGYCSKPDEC

ICRPGWQGRL CNECIPHNGC RHGTCSIPWQ CACDEGWGGL FCDQDLNYCT HHSPCKNGST

CSNSGPKGYT CTCLPGYTGE HCELGLSKCA SNPCRNGGSC KDQENSYHCL CPPGYYGQHC

EHSTLTCADS PCFNGGSCRE RNQGSSYACE CPPNFTGSNC EKKVDRCTSN PCANGGQCLN

RGPSRTCRCR PGFTGTHCEL HISDCARSPC AHGGTCHDLE NGPVCTCPAG FSGRRCEVRI

THDACASGPC FNGATCYTGL SPNNFVCNCP YGFVGSRCEF PVGLPPSFPW.

Ad.sDll4 gives rise to the over‐expression of the extracellular portion of Delta‐like ligand 4, which is sufficient to activate Notch signaling, nuclear translocation of NICD, and Hes/Hey upregulation in ECs.^14^

Viruses were titrated by end‐point dilution and purified using the AdEasy Virus Purification Kit (2 × 100).

### 2.2 In vitro angiogenesis assay

CHO cells were infected at a MOI of 100 and used to produce adenovirus‐conditioned media for 3 days. Normal human dermal fibroblast cells (NHDF, Promocell) were seeded onto coverslips for 3 days. Human dermal blood endothelial cells (HDBECs, Promocell) were then seeded on top of the fibroblasts and allowed to settle overnight. The addition of fibroblasts allowed for a continuously renewing layer of fibronectin and collagen to be synthesized, which supported spontaneous organization of ECs into tubule‐like structures.^15^ The cell culture media was then replaced with half volume of fresh media and half volume adenovirus‐conditioned media and changed every 2 days for 2 weeks, thus enabling us to test the functionality of the proteins produced via adenoviral‐mediated infection. After 2 weeks, the media was removed, and the cells were fixed with ethanol, stained with polyclonal rabbit anti‐VE‐Cadherin (5 μg/mL, ab33168, Abcam), and counterstained with DAPI. z‐stack images of 5 sections containing tubes from each coverslip were acquired, and image analysis was undertaken blinded using ImageJ.^16^ We counted number of endothelial tubes, measured tube segment length, and expressed sprout point and branch point number per unit area.

### 2.3 Hindlimb ischemia, gene transfer, and color speckle imaging

In accordance with the Animal (Scientific Procedures) Act (UK) of 1986 prepared by the Institute of Laboratory Animal Resources, C57Bl6 mice were subjected to surgical induction of unilateral hindlimb ischemia, as previously described (Couffinhal et al., 1998). Anesthesia was induced with 5% isoflurane vaporized in 100% oxygen at a rate of 1 L/min and maintained at 2% isoflurane. Body temperature was controlled throughout with a homeothermic blanket and rectal probe (Harvard Apparatus). The skin was epilated and sterilized using a chlorhexidine‐based solution. All mice were administered pre‐operative analgesic (buprenorphine at 0.05 mg/kg) and saline solution (0.9%NaCl at 40 mL/kg). An incision was cut in the groin region, and connective tissue was teased apart to expose the main artery supplying blood flow to the hindlimb. The nerve bundle was teased apart and the femoral artery was ligated in two sites above the epigastric branch and electrocoagulated in between, to induce ischemia to the left hindpaw. Immediately after ischemic induction, in all animals, 10^9^ pfu of each adenovirus was injected intramuscularly in the ischemic adductor. The superficial blood flow to both ischemic and non‐ischemic feet was measured using a color laser speckle imaging system (Moor FLIP2; Moor Instruments) in anesthetized animals with 1% isoflurane at days 0, 3, 7, 14, and 21 after induction of limb ischemia.^17^ The ratio of blood flow between the ischemic and contralateral foot was calculated to use as an index of percentage blood flow recovery.

### 2.4 Digital droplet PCR

At day 3 post surgery, tissue was recovered from some animals and lysed using Trizol with mechanical homogenization to extract RNA. 1 μg RNA was extracted, treated with DNAse, and transcribed to cDNA using PrimeScriptII (Takara). Per 25 μL reaction, 1 μL of transcript was added to 12.5 μL ddPCR Supermix for probes (Biorad), 2.5 μL Taqman probe (Life Technologies), and 9 μL water. Droplets were generated by adding the 25 to 70 μL Droplet oil (Biorad) using a QX200 droplet generator (Biorad). Amplification was carried out using standard Taqman protocols using a PCR thermal cycler (Biorad). Samples were analyzed using a QX100 droplet reader (Biorad). Thresholding was manually performed, based on negative and positive control results when automatic thresholding of values was not possible.

### 2.5 Immunofluorescence

**Figure 1:**
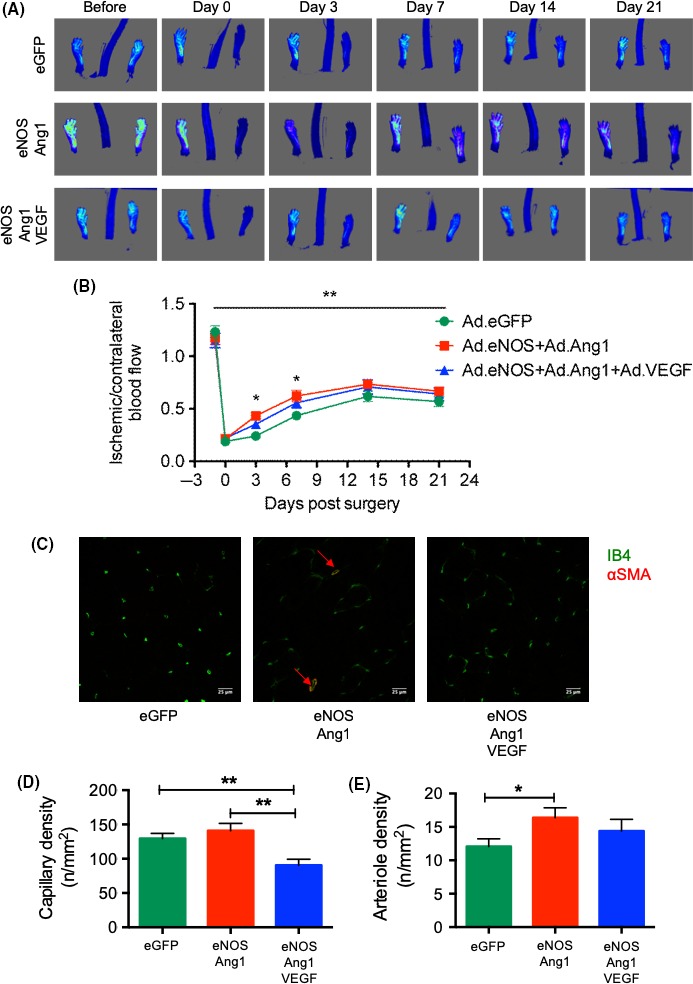
VEGF inhibits NO‐Tie‐mediated revascularization in C57Bl6 mice. (A) Laser speckle images from representative animals over the course of recovery. (B) Two‐way repeated measures ANOVA of the ischemic/contralateral ratio reveals that virus had a significant effect in the variation of recovery profiles (5.89% of total variance). This was particularly important at 3 and 7 days after surgery, when NO‐Tie mice (injected with Ad.eNOS+Ad.Ang1) had faster blood flow recovery compared with control mice (injected with Ad.eGFP), while there was no difference between control and NO‐Tie‐VEGF mice (injected with Ad.eNOS+Ad.Ang1 + Ad.VEGF). n = 12 mice/group. Two‐way repeated measures analysis of variance followed by Tukey's multiple comparison test: *P < 0.05, **P < 0.01. (C) Confocal images from representative muscle sections of ischemic adductors stained with isolectin B4 for endothelial cells (green) and α‐smooth muscle actin for vascular smooth muscle cells (red). Scale bar = 25 μm. (D) Capillary density is reduced in NO‐Tie‐VEGF mice when compared to both control and NO‐Tie mice. (E) Arteriole density is increased in NO‐Tie mice relative to control mice. n = 5‐6 mice/group. Kruskal‐Wallis test with α = 0.05 and Dunn's multiple comparison tests: *P < 0.05, **P < 0.01

At the experimental end‐point, mice were euthanized and the ischemic adductor muscles were dissected and embedded in O.C.T. compound in liquid nitrogen‐cooled isopentane. These muscles were sectioned and stained with IB4 and α‐SMA‐Cy3. Sections were imaged with a confocal microscope (Leica SPE), and counts of 30 random microscopic fields per mouse were averaged. Blood vessel profiles measuring around 5 μm in diameter stained only with IB4 were counted as capillaries, and those displaying a second cellular layer stained with SMA, surrounding the inner one, were counted as arterioles (for examples, see Figure 1C). Results were expressed as density of capillaries and arterioles per mm^2^.

## 3 RESULTS

The induction of unilateral hindlimb ischemia in 12‐week‐old male C57Bl6 mice resulted in an immediate decrease in perfusion to <20% of the pre‐surgery ratio (Figure 1A). Blood flow tended to recover up to 70% of the pre‐ischemia values in all animal groups by day 21 after surgery, reaching a peak by day 14 after ischemia (Figure 1B). Recovery between groups was not different at the final time point, when tissues were collected, stained (Figure 1C) and images obtained and analyzed for capillary (Figure 1D) and arteriole density (Figure 1E).

Intramuscular injection of adenovirus within the ischemic adductor muscle of mice, inducing the over‐expression of growth factors endothelial nitric oxide synthase (Ad.eNOS) and Angiopoietin 1 (Ad.Ang1), led to a more rapid improvement in the increase of blood flow in the ischemic hindpaw (Figure 1A), but no difference in the total recovery. This difference in recovery profiles was primarily attributable to a faster recovery of NO‐Tie mice (mice injected with Ad.eNOS+Ad.Ang1) compared to control mice (Ad.eGFP‐injected mice) (Figure 1B). Recovery of blood flow reached 64.5 ± 5.2% of baseline in NO‐Tie mice 14 days after ischemia, dipping slightly after this. Surprisingly, when mice were injected with Ad.eNOS+Ad.Ang1 + Ad.VEGF (NO‐Tie‐VEGF mice), there was no significant initial improvement in blood flow, and the shape of the recovery profile was not different than that of control mice (Figure 1B).

Analysis of muscle revascularization at 21 days after ischemia revealed that these early differences in blood flow, that were no longer present after day 7, were nonetheless accompanied by a marked change in the characteristics of the vascular network formed (Figure 1C). Capillary density was not increased in NO‐Tie mice compared to control mice, and it significantly decreased in NO‐Tie‐VEGF mice when compared with either control or NO‐Tie mice (Figure 1D). In contrast, arteriole density was significantly higher in NO‐Tie mice than control mice, but not altered in NO‐Tie‐VEGF mice (Figure 1E).

**Figure 2:**
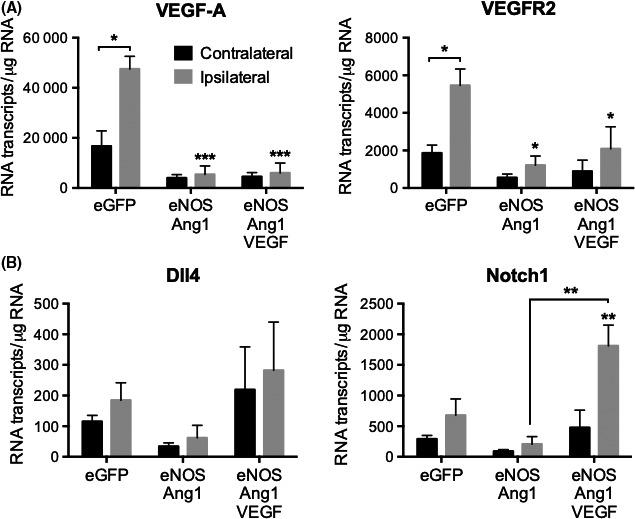
Increases in VEGF‐A/KDR and Notch1 signaling are concomitant with poor blood flux recovery. (A) VEGF‐A and KDR transcripts were significantly upregulated in the ischemic leg compared to the contralateral leg of control animals (*P < 0.05, two‐tailed paired t‐test) but this did not happen in the other animal groups. VEGF‐A and KDR transcripts were significantly lower in the ipsilateral adductors of NO‐Tie and NO‐Tie‐VEGF animals than on the ischemic adductors of control animals, although no differences were observed between them (***P < 0.001, ANOVA with Holm‐Sidak post‐test). (B) Although no differences were observed in Dll4 expression, expression of its receptor Notch1 was significantly upregulated in ischemic adductors of NO‐Tie‐VEGF mice when compared to NO‐Tie mice (**P < 0.005, ANOVA with Holm‐Sidak post‐test). (n = 4 animals/group)

In order to assess whether the unbalanced arteriole and capillary density observed are caused by a failure in the pathways of arterial specification due to increased VEGF signaling, we conducted a gene expression analysis using ddPCR (Figure 2).

In control mice, transcripts for endogenous VEGF and VEGFR2 were significantly upregulated 3 days after surgery in the ischemic leg, indicating an upregulation in this signaling pathway in response to hypoxia, as expected (Figure 2A). This was not the case in NO‐Tie and NO‐Tie‐VEGF mice, where the slight increase in transcript proportion of total RNA was not different from the contralateral leg, and significantly reduced when compared with control mice. This indicated that, even at this early time point, the severity of hypoxia and ischemia in NO‐Tie and NO‐Tie‐VEGF mice was reduced relative to control.

Thus, if increased VEGF signaling driving angiogenesis was not the cause of the imbalance observed in angiogenesis vs arteriolargenesis, maybe Notch signaling was causing this. While Dll4 was not significantly upregulated by ischemia or induced by NO‐Tie signaling, its receptor Notch 1 was upregulated in the ischemic adductor of NO‐Tie‐VEGF mice when compared to NO‐Tie mice (Figure 2B). This points in the direction of a push toward vessel maturation in NO‐Tie‐VEGF mice, that occurred far too early, compared to NO‐Tie mice, to have a positive impact in blood flow recovery.

**Figure 3:**
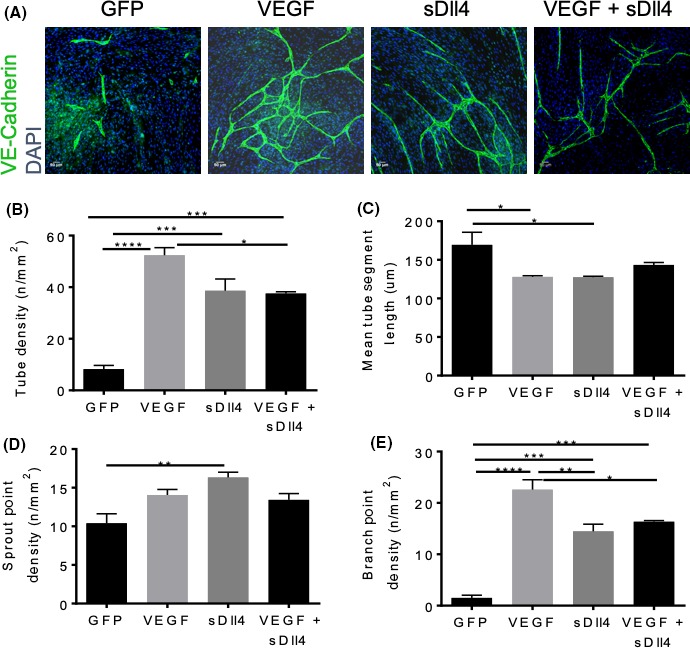
Ad.sDll4 increased sprouting but reduced branching. (A) HDBECs were co‐cultured on top of fibroblasts in an in vitro angiogenesis assay. They were treated with conditioned media for either Ad.eGFP, Ad.VEGF, Ad.sDll4, or Ad.VEGF + Ad.sDll4 every other day for 2 weeks (n = 3 experimental replicates, experiment was repeated three times). Cells were then fixed and stained for the endothelial junctional marker, VE‐Cadherin (green) and the nuclear marker, DAPI (blue). (B) Both sDll4 and VEGF + sDll4 resulted in a significantly higher tube density (sDll4: *P* = 0.0004, sDll4 + VEGF:* P* = 0.0005), than the control eGFP, yet shorter mean tube lengths (C). Treatment with sDll4 resulted in tubes with the most sprouts (16.33 ± 0.677 *P* = 0.0091); however, VEGF alone and in combination with sDll4 also increased this (D). Additionally, all three treatments increased the density of branches, with VEGF inducing the most and sDll4 alone resulting in slightly less than the combination treatment. One‐way ANOVA with a Bonferroni post‐test, with a 95% confidence interval. *P < 0.05, **P < 0.01, ***P < 0.001, ****P < 0.0001

We have previously shown that activation of Notch signaling by soluble Dll4 (sDll4) decreases vascular permeability, mainly due to an increased expression of VE‐Cadherin at intercellular junctions,^14^ and thus we used an adenovirus (Ad.sDll4) to promote early vessel maturation in the hindlimb ischemia mouse model, in order to assess its effect on recovery of blood flow. Firstly, to confirm that sDll4 was able to induce angiogenesis as well, we used an in vitro angiogenesis assay, where co‐cultured fibroblasts and ECs were treated with adenovirus‐conditioned media, stained, and imaged (Figure 3). In the unstimulated in vitro angiogenesis assay (eGFP), we observed singular tubes that were not connected to one another, whereas the tubes in the treatment conditions (VEGF, sDLL4, or both) were more interconnected (Figure 3A). Treatment with VEGF resulted in the highest density of tubes (*P* < 0.001 vs eGFP, Figure 3B). Treatment with sDll4 also resulted in a significantly higher density of tubes than eGFP but, when VEGF was given in combination with sDll4, the density of tubes was very similar to that of sDll4 alone. Conversely, mean tube segment length for all experimental conditions decreased. When given alone, VEGF and sDll4 led to a decrease in mean tube segment length but, when given in combination, tube segment length was slightly longer and was not different from control (Figure 3C). Sprout density was increased above eGFP levels when treated with VEGF, but sDll4 alone resulted in the highest sprout density (Figure 3D). When cells were treated with VEGF + sDll4, this increase in sprout density was attenuated. Branch point density was also significantly increased above eGFP for all conditions (Figure 3E). We concluded that VEGF was the most efficient in promoting angiogenesis and that, when sDll4 was given alone, or in combination with VEGF, it attenuated this increase.

**Figure 4:**
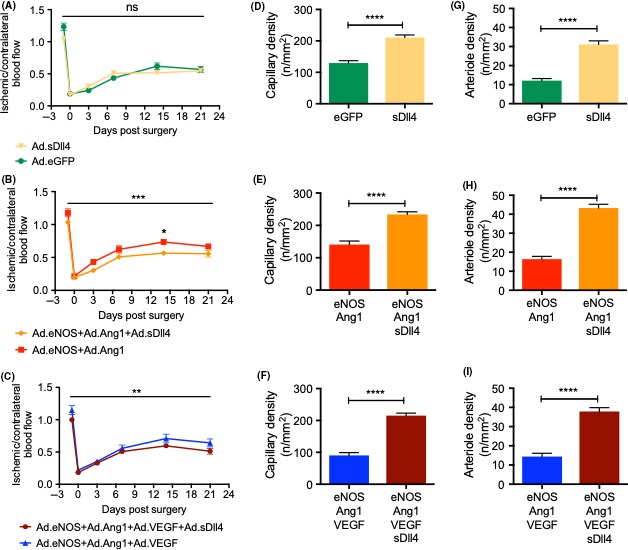
Ad.sDll4 impairs recovery of blood flux in NO‐Tie mice, even when paralleled by increased angiogenesis and arteriolargenesis. (A) Ad.sDll4 had no effect on recovery of blood flux after ischemia when compared to control mice but (B) a significant effect in the variation of recovery profiles was observed when this was added to NO‐Tie mice (3.22% of total variance, ***P = 0.0004). (C) Addition of Ad.sDll4 further worsened the outcome of NO‐Tie‐VEGF mice (1.80% of total variance, **P = 0.0070). Two‐way ANOVA with Sidak's post‐test: *P < 0.05, n = 12 mice/group. In all animal groups, addition of Ad.sDll4 led to an increase in both capillary (D‐F) and arteriole densities (G‐I). n = 5‐6 mice/group. Two‐tailed Mann‐Whitney test with 95% confidence interval: ****P < 0.0001

The role of Notch signaling in driving assembly of adherens junctions and regulating endothelial barrier integrity has recently been described.^14^, ^18^ These are molecular pathways involved in vessel maturation and, thus, precursors to arteriolargenesis. So, we assessed the effect of sDll4 in NO‐Tie‐induced recovery of blood flow after ischemia (Figure 4). We found that adenoviral‐mediated sDll4 over‐expression in the ischemic adductor had no effect in recovery of blood flow after ischemia when compared to control mice (Figure 4A). However, sDll4 significantly impaired the positive effect in blood flux of NO‐Tie recovery after ischemia, specifically at day 14 after surgery (Figure 4B). Surprisingly, addition of Ad.sDll4 further worsened the outcome of NO‐Tie‐VEGF mice as well (Figure 4C). Moreover, sDll4 addition significantly increased both capillary and arteriole densities in all animal groups (Figure 4D‐I), which points to a mismatch between metabolic demands and blood vessel densities, leading to lack of blood flow control at the vascular network level.

## 4 DISCUSSION

Similar to its effects on the rat mesentery assay,^12^ NO‐Tie had a pro‐arteriolargenic effect in a unilateral murine model of hindlimb ischemia, without impacting on angiogenesis, giving rise to an accelerated recovery of blood flow (Figure 1). Even though this beneficial effect was not sustained at later time points, it happened even in the presence of a significantly reduced endogenous VEGF signaling in the ipsilateral leg, compared to control mice (Figure 2A).

In the rat mesentery assay, VSMC recruitment or differentiation is not inhibited by over‐expression of VEGF‐A.^13^ Indeed, NO‐Tie‐VEGF mice were still able to increase arteriolargenesis; however, this occurred at the expense of reduced capillary density (Figure 1C,D). It seems that a source of exogenous VEGF moves revascularization toward earlier vessel maturation, thereby disturbing the balance of the different vessel types needed for adequate control of blood flow. It is well established that over‐expression of VEGF on its own does not have therapeutic utility for the treatment of peripheral ischemia,^3^ but the idea that VEGF signaling is actually deleterious to revascularization capable of improving recovery of blood flow in this model of disease has only recently been proposed.^19^ Several studies have proposed that the source of VEGF is important to its function in arteriogenesis and vessel maturation. VEGFR2 trafficking within ECs through endocytic vesicles regulates arterial morphogenesis 20 and requires the cytoplasmic domain of neuropilin 1.^20^ Moreover, ablation of the scaffold protein synectin expression in ECs, but not VSMCs, resulted in impaired arteriogenesis in adult mice 21 and this phenotype was rescued upon endothelial‐specific knockout of phosphotyrosine phosphatase 1b.^22^ Thus, it appears that VEGF signaling and VEGFR2 internalization in specific endosomes within ECs may be required for arteriolargenesis. However, when the source of VEGF comes from the ischemic muscle, the subsequent recruitment of VSMCs may prevent further sprouting angiogenesis and lead to premature arterialization of the newly formed vasculature in the ischemic muscle.

Therefore, we sought some molecular cues to help us understand this process and found that one of the genes involved in arterial specification, Notch 1, was upregulated in NO‐Tie‐VEGF mice, when compared to NO‐Tie mice (Figure 2B). Jagged 1/Notch 1 signaling pathway has been implicated in endothelial‐mesenchymal transition—it is activated in microvascular ECs in the bleomycin‐induced model of pulmonary fibrosis in rats, where it may induce α‐smooth muscle actin expression via a non‐canonical pathway involving NFκB.^23^ Activation of Notch 1 signaling has also been found in brain arteriovenous malformations in humans.^24^ Furthermore, molecular alterations in Dll4‐Hey2 signaling are associated with VSMC hypertrophy and hyperplasia in varicose veins.^25^ Recent gene expression signatures associated with Notch ligands Dll4 and Jagged 1 in plaque material from PAD patients have been associated with disease progression.^26^ Adult Dll4^+/−^ mice have increased angiogenesis, decreased arteriolar density, more severe tissue ischemia, and poor blood flow recovery after femoral artery occlusion. They also present increased plasma leakage, decreased flow‐induced outward remodeling, increased arteriolar contractility, and impaired arteriolar responses to shear‐stress and vasoactive molecules.^27^

In the past, a different Ad.sDll4 has been used as a decoy, inhibiting Notch signaling, downregulating expression of Hes, and giving rise to a disorganized and non‐perfused capillary network in a model of hindlimb ischemia.^28^ However, we have found that sDll4 is sufficient to upregulate Hes expression and activate Notch signaling, tightening the endothelial barrier *in* vitro and in vivo.^14^ Here, we find that sDll4 is sufficient to induce an angiogenic response. However, in an in vitro angiogenesis assay, the resulting tubule network has a different topology than the one elicited by VEGF. The EC tubes formed had more blind‐ends and were less interconnected than those formed by VEGF alone. When VEGF and sDll4 were simultaneously applied, the aspect of the network resembled that of sDll4‐only stimulation (Figure 3). Given the rising interest and often conflicting data on the role of Dll4/Notch signaling in promoting vessel maturation 27, 28, 29, 30 and our own experience researching the role of Dll4 in regulating vascular permeability and intercellular communication,^14^ we also tested how Dll4/Notch signaling affected recovery of blood flow after ischemia. We postulated that the deleterious effect of VEGF could possibly be explained by the activation of Notch in the ischemic muscle, and that we could be able to recapitulate this phenotype using sDll4. We observed a phenotype of unproductive angiogenesis and arteriolargenesis elicited by sDll4 (Figure 4D‐I). The significantly denser vascular network had no effect on recovery from ischemia (Figure 4A) or a deleterious effect on the NO‐Tie‐mediated recovery of blood flow after ischemia (Figure 4B). sDll4 further worsened the outcome of NO‐Tie‐VEGF mice (Figure 4C). This suggests that sDll4 may have acted to prevent vessel maturation at earlier time points, leading to a denser, but less efficient vascular network with reduced functional capacity. It may be that forcing a pathway of arterial specification earlier on in the healing process is deleterious, since it may bring about the uncoupling of excitation‐conduction in newly formed arterioles. It also points to the concept that, if the vascular network formed does not match the metabolic demands of the tissue, then unproductive arteriolargenesis is as ineffective to recovery from ischemia as unproductive angiogenesis.

The experiments described here use a mouse model of ischemic revascularization that results in relatively rapid recovery. Given that the temporal administration of the adenovirus (AdV) here was all done immediately after the ischemia, it is quite possible that this model is neither representative of the human disease, where ischemia arises gradually, and that the AdV is given at the wrong time—it would be interesting to determine whether the time of AdV administration is critical for the positive effects of the different AdV combinations.

Together, these results suggest that the negative effect of exogenous VEGF in NO‐Tie mice recovery from blood flow may be paralleled by, but is not synergistic with, Notch1 activation, and that the quality of the revascularization in terms of the balance between the temporal and spatial development of arterioles vs capillaries is critical for effective recovery from ischemia.

## AUTHORS' CONTRIBUTIONS

Experiments were carried out by MJM, RB, AVB, and FR. Data was collated, checked, and analyzed by MJM, RB, AVB, FR, and DOB. Experimental design was carried out by all authors. MJM drafted, and all authors critiqued and contributed to the final manuscript.
